# Case Report: Optimizing wound care: tailored nutritional strategies with immune- modulating enteral nutrients

**DOI:** 10.3389/fnut.2026.1758701

**Published:** 2026-03-13

**Authors:** Fiji Antony, Wafaa Ayesh

**Affiliations:** 1 Department of Dietetics, NMC Speciality Hospital, Dubai, United Arab Emirates; 2 Al Tadawi Specialty Hospital, Dubai, United Arab Emirates

**Keywords:** energy, enteral nutrition, medical nutrition therapy, multidiscipliary team, optimum nutrition, pressure ulcers, protein, tube feeding

## Abstract

**Background:**

Adequate nutrition and hydration are essential for maintaining skin integrity and supporting tissue repair in patients with pressure injuries. Current ESPEN guidelines emphasize the role of individualized nutritional therapy, including optimized energy and protein delivery and selected conditionally essential nutrients such as arginine, glutamine, and *β*-hydroxy-β-methylbutyrate (HMB), as part of a multidisciplinary approach to pressure ulcer management. This case series describes dietitian-led personalized nutritional interventions in critically ill, long-term hospitalized patients with advanced pressure ulcers.

**Case summary:**

This descriptive case series reports four adult patients receiving long-term enteral nutrition in an acute or long-term care setting. Each case illustrates individualized nutritional assessment and intervention tailored to metabolic stress, nutritional risk, wound burden, and tolerance. Nutritional strategies included early initiation and optimization of enteral feeding, progressive achievement of energy and protein targets, glycemic control, and selective use of immunonutrition. Over prolonged follow-up, all patients demonstrated improvement and eventual healing of pressure ulcers within a comprehensive multidisciplinary care bundle. This series highlights the practical application of personalized nutrition in complex clinical scenarios rather than establishing causal efficacy.

**Exclusion criteria:**

Patients were excluded from this case series if they met any of the following conditions: (1) Age below 18 years, (2) Pregnancy or lactation, (3) Patients receiving exclusive parenteral nutrition, (4) Presence of terminal illness or receiving palliative/end-of-life care, (5) Hemodynamic instability preventing initiation or continuation of enteral nutrition, (6) Pre-existing metabolic disorders that could significantly confound nutritional outcomes (e.g., inborn errors of metabolism), (7) Incomplete medical or nutritional records, and (8) Patients with anticipated length of stay are insufficient to assess nutritional intervention outcomes.

**Inclusion criteria:**

Patients were included in this case series if they met the following criteria: (1) Adult patients aged 18 years and above, (2) Admitted to an acute or long-term care setting requiring nutritional intervention, (3) Presence of medical conditions associated with increased nutritional risk, such as critical illness, neurological impairment, psychiatric disorders, or impaired wound healing, (4) Patients who received individualized nutritional assessment and intervention, including enteral nutrition, (5) Availability of complete medical, nutritional, and clinical outcome records, and (6) Patients with a length of hospital stay sufficient to allow assessment of nutritional intervention outcomes.

**Conclusion:**

This case series underscores the importance of individualized, dietitian-led nutritional therapy integrated within multidisciplinary care for patients with advanced pressure injuries. Optimized nutritional delivery was associated with improved nutritional status and wound healing progression; however, causal inference is limited by the observational design. Prospective studies using standardized wound assessment tools are warranted to clarify the independent contribution of targeted nutritional interventions.

## Introduction

Malnutrition and inadequate nutrient intake are well-established risk factors for the development and delayed healing of pressure ulcers (1). In critically ill patients, the catabolic stress response induces systemic inflammation, muscle protein breakdown, and impaired immune function, increasing the risk of infection, prolonged hospitalization, and mortality (2, 3).

Historically, nutrition support in critical care focused on providing energy to prevent starvation. Contemporary practice has shifted toward therapeutic nutrition, aiming to attenuate metabolic stress, preserve lean body mass, modulate inflammation, and support tissue repair (4–6). Accordingly, modern enteral formulations increasingly include bioactive nutrients designed to support immune function, oxidative balance, and wound healing (7).

ESPEN and ASPEN guidelines recommend early enteral nutrition when feasible, individualized energy and protein targets, careful glycemic control, and selective use of conditionally essential nutrients in patients with impaired wound healing (6, 7). Dietitians play a central role in translating these recommendations into individualized clinical practice.

The objective of this case series is to describe the implementation and outcomes of dietitian-led personalized enteral nutrition strategies in critically ill patients with advanced pressure ulcers within a multidisciplinary care framework.

## Methodology

This descriptive case series included adult patients receiving long-term enteral nutrition in an acute or long-term care facility. All patients were managed by a multidisciplinary team led by an intensivist and including clinical dietitians, nurses, wound care specialists, physiotherapists, occupational therapists, and speech and language therapists.

### Nutritional assessment

Nutritional risk was assessed using the Nutritional Risk Screening 2002 (NRS-2002) tool. A comprehensive nutrition-focused physical examination (NFPE) was conducted by a clinical dietitian, with documentation of muscle wasting and subcutaneous fat loss in patients identified as severely malnourished. The same dietitian team conducted assessments throughout follow-up to ensure consistency.

Body weight was measured weekly using a calibrated portable bed scale. Energy and protein requirements were estimated using weight-based equations in accordance with international guidelines (ESPEN/ASPEN), as indirect calorimetry was not routinely available.

### Nutritional intervention

Enteral nutrition was initiated early when clinically feasible and advanced gradually to target energy and protein goals to minimize feeding intolerance and metabolic complications. Protein delivery was prioritized to support wound healing, immune function, and preservation of lean body mass.

Selective immunonutrition supplementation was introduced in patients with severe malnutrition or impaired wound healing, using a standardized formulation containing glutamine, arginine, and calcium *β*-hydroxy-β-methylbutyrate (Ca-HMB). Each 24-g sachet provided:

Glutamine: 7 g.

Arginine: 7 g.

Ca-HMB: 1.5 g.

Supplementation was individualized based on clinical condition, tolerance, and ongoing reassessment, and was reviewed regularly by the dietitian in collaboration with the medical team.

### Feeding delivery and monitoring

Continuous enteral feeding was delivered using calibrated feeding pumps. Tolerance was assessed every 4–6 h, including gastric residual volumes, gastrointestinal symptoms, and abdominal examination. Feeding regimens were adjusted in response to intolerance using established protocols.

### Multidisciplinary care bundle

Nutritional therapy was delivered within a standardized care bundle including:

Head-of-bed elevation >30°.

Glycemic control (140–180 mg/dL).

Pressure-relieving mattresses.

Scheduled repositioning.

Standardized wound care.

Deep venous thrombosis prophylaxis.

## Case descriptions

All four cases demonstrated progressive improvement in pressure ulcer status over prolonged follow-up while receiving individualized nutritional therapy within a multidisciplinary framework. Nutritional targets were adjusted according to tolerance, metabolic stress, and evolving clinical status. Improvements in nutritional status and wound healing occurred alongside concurrent medical, nursing, and wound care interventions.

### Case 1

A 73-year-old female patient with a history of a cerebral vascular accident (CVA) caused by an embolism in the left middle cerebral artery, along with comorbid conditions including diabetes mellitus (DM), hypertension (HTN), and coronary artery disease (CAD). The patient underwent a tracheostomy and was provided with a percutaneous endoscopic gastrostomy (PEG) for enteral feeding. She was transferred to this hospital with a grade 4 pressure ulcer, as assessed by the Yarkony-Kirk scale. Over a period of 7 days, the nutritional goal was met, providing 1.2 grams of protein and 20–25 kcal per kilogram of body weight from a disease- specific diabetic formula. Additionally, the nutrition was supplemented with HMB, Arginine, and Glutamine within the first week of admission. The pressure ulcer showed progressive healing over the following 11 months. Biochemical lab results revealed a slight decrease in blood urea nitrogen (BUN) over 12 months, with stable hemoglobin and serum albumin levels.

### Case 2

A 30-year-old female with a complex medical history, including bipolar disorder, progressive cognitive and motor decline, hypothyroidism (currently treated), and a past case of COVID-19 pneumonia, presents with recurrent urinary tract infections (UTIs) and multiple pressure ulcers. She started enteral feeding using a silicone fine-bore nasogastric tube. Upon examination, she is found to have severe malnutrition (NRS 2002), with a BMI of 15.82, and as per nutrition focused physical finding, significant muscle wasting, and a loss of subcutaneous fat. A customized nutrition plan is developed to prevent refeeding syndrome, with a gradual increase in caloric intake over four to 8 weeks while monitoring electrolytes (phosphate, magnesium, potassium) and feed tolerance. Enteral nutrition begins with a standard formula at a low infusion rate, progressively increased to meet optimal nutritional delivery over four to 8 weeks. This targets an energy intake of 50 kcals per kg body weight and 2.8 grams of protein per kg body weight at peak tolerance, along with additional nutrients to support wound healing (HMB, Arginine, Glutamine). After 10 months, significant.

Improvements are seen, including an increase in body weight (BMI 18.11) and the healing of pressure ulcers, which are fully healed by 12 months.

### Case 3

A 38-year-old male, diagnosed with hypoxic–ischemic brain damage following cardiac arrest, underwent tracheostomy and presented with recurrent pneumonia and grade 4 pressure ulcers on his sacrum and leg, requiring PEG feeding. Upon admission, the patient showed signs of malnutrition, with a BMI of 17.57 (NRS 2002), and nutrition focused physical findings with significant fat loss in the triceps and subcutaneous fat over the ribs. Muscle wasting was visible in the shoulder, scapula, thigh, and calf, but there were no signs of fluid retention. Nutritional intervention was initiated promptly, starting with a gradual increase in the rate of a standard.

polymer formula, supplemented with specialized nutrients aimed at wound healing, including HMB, Arginine, and Glutamine. The patient reached peak nutritional tolerance at 38 kcal per kg of body weight and 1.6 gm of protein per kg of body weight. Over the course of a year, there was notable improvement in the pressure ulcers, alongside a gradual increase in body weight.

### Case 4

**Figure 1:**
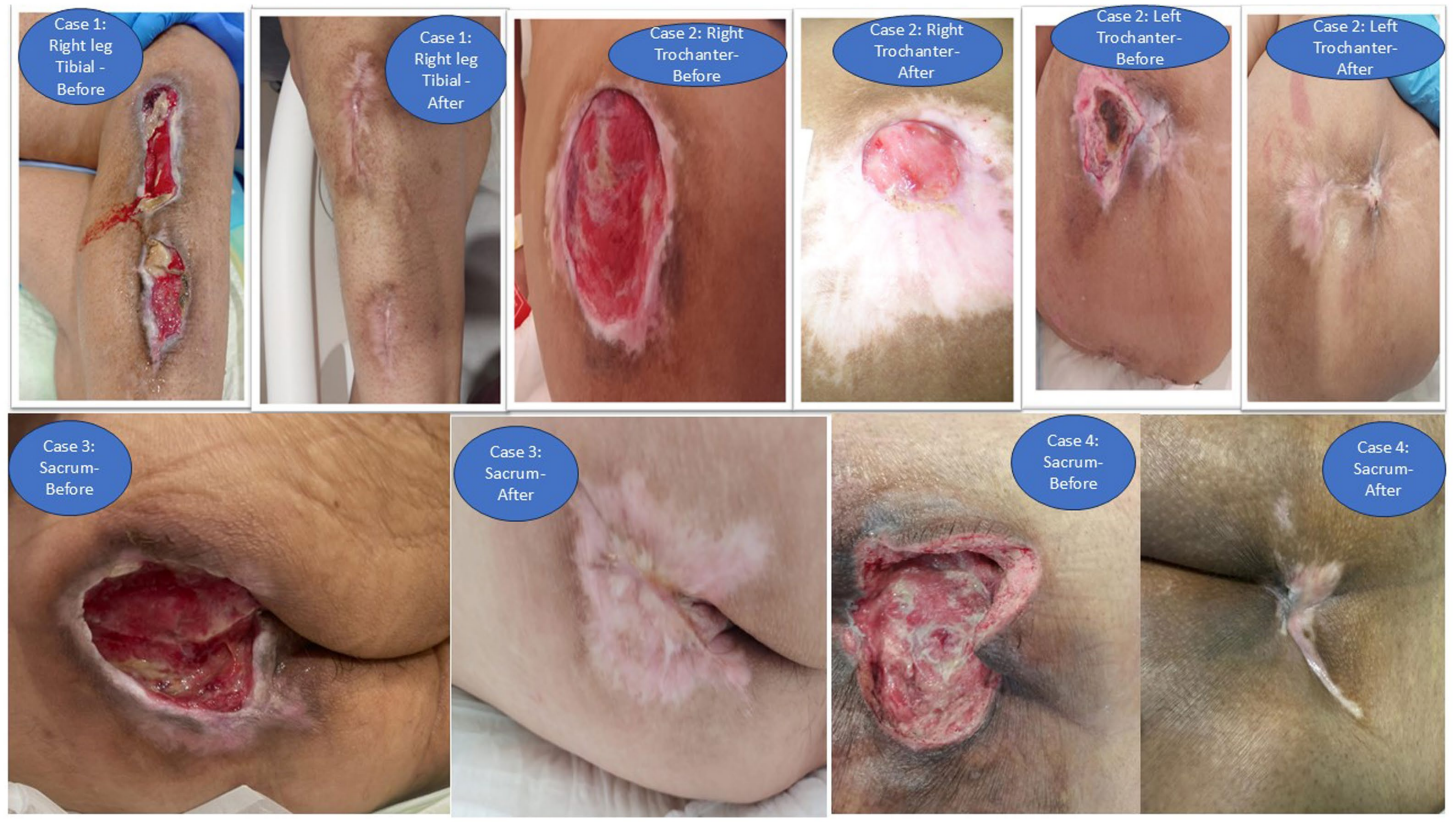
Proposed framework for tailored nutritional strategies in wound care, highlighting the role of immune-modulating enteral nutrients in supporting inflammation control, tissue repair, collagen synthesis, and overall wound healing outcomes.

A total of 85-year-old female with a history of CVA (right hemiplegia), COVID-19, Alzheimer’s, and recurrent UTIs. She was tracheostomized and receiving PEG tube feeding. Upon admission, her BMI was 23.83, and she had a grade 4 pressure ulcer on her left gluteus. A standard polymer formula and specialized nutrition (including HMB, Arginine, and Glutamine) were used to meet her nutritional goals. The target nutrition plan aimed for 33 kcal per kg of body weight and 1.8 g of protein per kg of body weight. However, after 8 months, the patient began experiencing recurring intolerance to the formula, with vomiting and diarrhea. Consequently, the formula was switched to a hydrolyzed one. The new plan adjusted her energy intake to 27 kcal per kg of body weight and protein to 1.6 g per kg of body weight. After 10 months, the pressure ulcers healed, and her nutrition plan was updated to 29 kcal per kg of body weight and 1.3 g of protein per kg of body weight (Figure 1).

## Discussion

Adequate energy, protein, and micronutrient intake is fundamental for tissue repair, particularly in patients with advanced pressure injuries and prolonged critical illness. Nutritional prescriptions should be individualized according to illness severity, metabolic stress, organ function, and wound burden rather than applying uniform targets.

In this case series, dietitian-led nutritional strategies focused on early enteral nutrition, progressive achievement of energy and protein goals, and selective immunonutrition supplementation within a multidisciplinary care bundle. Enteral nutrition was preferred whenever feasible, consistent with current guidelines supporting gut integrity and reduced infectious risk compared with parenteral nutrition.

Importantly, pressure ulcer healing observed in this series reflects multimodal management, including optimized nutrition, pressure off-loading, glycemic control, infection management, and standardized wound care. While improved nutritional delivery was temporally associated with wound healing progression, causal inference cannot be established due to the observational design and concurrent interventions.

The use of arginine, glutamine, and HMB was guided by evidence suggesting potential benefits in collagen synthesis, immune modulation, and muscle protein metabolism. However, ongoing debate remains regarding the role of glutamine in critically ill populations, underscoring the need for careful patient selection and monitoring.

Limitations include the small sample size, lack of standardized quantitative wound measurements, and absence of a comparator group. Nevertheless, this series provides real-world insight into the practical implementation of personalized nutrition in complex patients and highlights the central role of dietitians within multidisciplinary teams (Table 1).

**Table 1:** Key immune-modulating enteral nutrients, their mechanisms of action, and clinical benefits in wound healing management.

Parameter	Oct 2022	Dec 2022	Jun 2023	Aug 2023	Oct 2023	Nov 2023
Case 1						
Body weight (kg)	72.5	70	65.5	69	64	64
Enteral feed volume (mL/day)	1,100	1,300	1,300	1,300	1,200	1,200
Total energy intake (kcal/day)	1,320	1,498	1,498	1,498	1,378	1,440
Total protein intake (g/day)	66	95.6	95.6	95.6	89.6	72
Body mass index (kg/m^2^)	26.63	25.71	24.06	25.34	23.51	23.51
HbA1c (%)	9.7	NA	10	NA	NA	NA
Serum albumin (g/dL)	3.57	2.91	3.57	3.68	3.56	NA
Hemoglobin (g/dL)	14.8	11.1	10.8	13.2	13.1	13.7
Blood urea nitrogen (mg/dL)	132	36	43	51	47	NA
Pressure ulcer/wound status	Grade 4	Healing	Healing	Healing	Healing	Healed
Case 2						
Body weight (kg)	38	38	39	40	43.5	43.5
Enteral feed volume (mL/day)	810	1,400	1,400	1,400	1,400	1,300
Total energy intake (kcal/day)	1,260	1978	1978	1978	1978	1950
Total protein intake (g/day)	53.6	106.16	106.16	106.16	106.16	82.94
Body mass index (kg/m^2^)	15.82	15.82	16.23	16.65	18.11	18.11
HbA1c (%)	4.7	NA	NA	NA	NA	NA
Serum albumin (g/dL)	4.38	3.71	3.63	4.12	3.72	NA
Hemoglobin (g/dL)	13.1	11	12.3	11.2	12.3	12.6
Blood urea nitrogen (mg/dL)	12	24	20	21	17	NA
Pressure ulcer/wound status	Grade 4	Healing	Healing	Healing	Healing	Healed
Case 3						
Body weight (kg)	49	50	52	52.5	52.5	52.5
Enteral feed volume (mL/day)	1,000	1,400	1,500	1,500	1,300	1,300
Total energy intake (kcal/day)	1,500	1987	2,128	2,128	1950	1950
Total protein intake (g/day)	63.8	106.2	112.54	112.5	82.94	82.94
Body mass index (kg/m^2^)	17.57	17.93	18.65	18.82	18.82	18.82
HbA1c (%)	NA	NA	NA	NA	NA	NA
Serum albumin (g/dL)	NA	4.45	NA	NA	NA	NA
Hemoglobin (g/dL)	13.4	14	11.5	11	NA	NA
Blood urea nitrogen (mg/dL)	NA	22	NA	NA	NA	NA
Pressure ulcer/wound status	Grade 4	Healing	Healing	Healing	Healed	Healed
Case 4						
Body weight (kg)	58	60	63	62	63	63
Enteral feed volume (mL/day)	1,100	1,400	1,400	1,200	1,200	1,200
Total energy intake (kcal/day)	1,650	1978	1978	1,678	1,678	1800
Total protein intake (g/day)	70.18	106.16	106.16	97.1	97.1	81
Body mass index (kg/m^2^)	23.83	24.65	25.89	25.48	25.89	25.89
HbA1c (%)	5.8	NA	NA	5.7	NA	NA
Serum albumin (g/dL)	4.1	3.24	3.61	3.66	3.67	NA
Hemoglobin (g/dL)	14.2	10.4	10.9	11.5	12.2	NA
Blood urea nitrogen (mg/dL)	15	15	31	29	18	NA
Pressure ulcer/wound status	0	Grade 4	Healing	Healing	Healing	Healed

## Clinical significance

This case series highlights the essential role of nutritional care in wound healing, especially for bedridden patients with multiple health conditions (8, 9). The study shows that personalized enteral feeding can enhance nutritional status and support wound recovery (10, 11). Additionally, the use of specialized supplements like Arginine, glutamine, and HMB highlights the significance of focused nutritional interventions in promoting healing and overall patient health (12, 13). In conclusion, the findings stress the importance of a holistic, patient-focused approach to nutritional care in managing complex wounds and improving clinical results (14–16).

## Conclusion

This case series illustrates the application of individualized, dietitian-led enteral nutrition strategies within multidisciplinary care for patients with advanced pressure injuries. Optimized energy and protein delivery, combined with selective immunonutrition and close monitoring, was associated with improvements in nutritional status and wound healing over time. Given the observational nature of the study, future prospective research using standardized wound assessment tools and controlled nutritional protocols is needed to clarify the independent effects of targeted nutritional interventions.

## Data Availability

The raw data supporting the conclusions of this article will be made available by the authors, without undue reservation.
